# The expression of the formin Fhod3 in mouse tongue striated muscle

**DOI:** 10.1247/csf.24044

**Published:** 2024-10-10

**Authors:** Hikaru Nakagawa, Yohko Kage, Ayako Miura, Hikmawan Wahyu Sulistomo, Sho Matsuyama, Yoshihiro Yamashita, Ryu Takeya

**Affiliations:** 1 Department of Pharmacology, Faculty of Medicine, University of Miyazaki, Miyazaki 889-1692, Japan; 2 Division of Oral and Maxillofacial Surgery, Department of Medicine of Sensory and Motor Organs, Faculty of Medicine, University of Miyazaki, Miyazaki 889-1692, Japan; 3 Department of Pharmacology, Faculty of Medicine, Universitas Brawijaya, Malang 65145, Indonesia

**Keywords:** actin, formin, sarcomere, striated muscle

## Abstract

The sarcomere is the contractile unit of striated muscle and is composed of actin and myosin filaments. There is increasing evidence to support that actin assembly mediated by Fhod3, a member of the formin family of proteins, is critical for sarcomere formation and maintenance in cardiac muscle. Fhod3, which is abundantly expressed in the heart, localizes to the center of sarcomeres and contributes to the regulation of the cardiac function, as evidenced by the fact that mutations in Fhod3 cause cardiomyopathy. However, the role of Fhod3 in skeletal muscle, another type of striated muscle, is unclear. We herein show that Fhod3 is expressed in the tongue at both mRNA and protein levels, although in smaller amounts than in the heart. To determine the physiological role of Fhod3 expressed in the tongue, we generated embryos lacking Fhod3 in the tongue. The tongue tissue of the Fhod3-depleted embryos did not show any significant structural defects, suggesting that Fhod3 is dispensable for normal development of the mouse tongue. Unexpectedly, the immunostaining analysis revealed the absence of specific sarcomeric signals for Fhod3 in the wild-type tongue when compared to the Fhod3-depleted tongue as a negative control, despite the use of antibodies that had previously been validated by immunostaining of heart tissues. Taken together, although Fhod3 protein is expressed at a significant level in the tongue, Fhod3 in the tongue does not appear to exhibit the same sarcomeric pattern as observed in the heart, suggesting a different role for Fhod3 in the tongue muscles.

## Introduction

Striated muscles have a highly ordered ultrastructure consisting of sarcomeres, which are contractile units containing thick myosin-based filaments and thin actin-based filaments (Henderson *et al.*, 2017). Upon excitation of the muscle cell, myosin heads bind to actin to form cross-bridges, and then actin filaments slide into a lattice of myosin filaments to contract the muscle. These primary structural characteristics of sarcomeres are conserved between cardiac and skeletal muscles, although considerable differences exist in electrophysiological properties, contractile functions, development, and maintenance processes. A proper understanding of the similarities and differences, as well as the underlying molecular mechanisms, will provide insights into the comprehensive mechanisms governing the contractile function of muscles and the molecular pathology of various muscle diseases.

Mutations in sarcomeric proteins have been associated with various muscle diseases (Henderson *et al.*, 2017). One of the major categories of sarcomeric proteins is actin-related protein. Mutations in these proteins are associated with myopathy in the skeletal muscles and cardiomyopathy in the cardiac muscles. It has recently become evident that proteins that regulate actin dynamics and actin filament turnover, as well as the structural components of thin actin filaments, are implicated in the pathogenesis of both myopathy and cardiomyopathy (Ono, 2010). For example, mutations in the actin depolymerization factors Cofilin2 and CAP2, cause skeletal and cardiac myopathy, respectively (Agrawal *et al.*, 2007; Aspit *et al.*, 2019). Lmod3, a skeletal isoform of actin polymerization factor, and its cardiac form Lmod2 also cause skeletal and cardiac myopathy, respectively (Ahrens-Nicklas *et al.*, 2019; Yuen *et al.*, 2014), similar to nebulin, an actin filament stabilizer in skeletal muscle, and its cardiac form nebulette (Pelin *et al.*, 1999; Purevjav *et al.*, 2010). Thus, the proper regulation of actin dynamics in the sarcomere is required for the striated muscle function.

The formin protein family is a key regulator of actin dynamics. Formin family proteins are structurally characterized by the presence of formin homology domains (FH) 1 and 2, and play pivotal roles in actin filament assembly in a variety of cellular processes (Chesarone *et al.*, 2010; Randall and Ehler, 2014). In mammals, the formin family is classified into 7 subfamilies with 15 members (Schönichen and Geyer, 2010). The Fhod subfamily includes 2 mammalian members, Fhod1 and Fhod3. Fhod1 (formerly designated as Fhos), the founder of the Fhod subfamily, is abundantly expressed in non-muscle cells, including endothelial and hematopoietic cells, and is involved in the formation of stress fibers in non-muscle cells (Takeya *et al.*, 2008; Takeya and Sumimoto, 2003). Another member of the Fhod subfamily, Fhod3, was originally identified as a homolog of Fhos and was designated as Fhos2 (Kanaya *et al.*, 2005). Fhod3 is highly expressed in the heart and it plays an essential role in the assembly of actin into sarcomeres in cardiomyocytes (Iskratsch *et al.*, 2010; Taniguchi *et al.*, 2009). Subsequent analyses using conventional and conditional Fhod3 knockout mice corroborated the physiological importance of Fhod3 at the whole-body level (Kan-o *et al.*, 2012; Matsuyama *et al.*, 2018; Ushijima *et al.*, 2018). Finally, a human genome analysis revealed that human mutations in Fhod3 indeed cause hypertrophic cardiomyopathy (Ochoa *et al.*, 2018), indicating the critical role of Fhod3 in cardiac muscle. However, the role of Fhod3 in skeletal muscle remains largely unknown.

Two Fhod isoforms are expressed in mammals, whereas a single Fhod protein is expressed in both flies and worms. The *Drosophila* Fhod protein is implicated in actin assembly in *Drosophila* indirect flight muscles (Shwartz *et al.*, 2016), and the *C. elegans* Fhod protein is involved in the development of body wall muscles of *C. elegans* (Sundaramurthy *et al.*, 2020). These lines of evidence suggest a possible role for Fhod3 in mammalian skeletal muscle.

In the present study, among the various types of skeletal muscles, we focused on the tongue muscle, which, like cardiac muscle, does not have a bone support system. A biochemical analysis revealed that Fhod3 is expressed in the tongue, albeit at a lower level than that in the cardiac muscle. The distribution of Fhod3 in tongue striated muscle did not appear to exhibit a sarcomeric pattern as observed in cardiac muscle, thus suggesting that Fhod3 may possess a function that is specific to skeletal muscles. These findings suggest the existence of different regulatory mechanisms for actin dynamics in different types of striated muscles.

## Materials and Methods

### Mice

Mice heterozygous for the constitutive null Fhod3 allele (*Fhod3^+/–^*) (Accession No. CDB0598K: https://large.riken.jp/distribution/mutant-list.html) were generated by replacing exon 1 with *lacZ* as described previously (Kan-o *et al.*, 2012). Transgenic *Fhod3^Tg(α-MHC-Fhod3CM)^* mice expressing wild-type Fhod3 of the cardiac muscle isoform under the control of the α-myosin heavy chain (α-MHC) promoter were generated as described previously (Matsuyama *et al.*, 2018). For deletion of Fhod3 in the tongue, *Fhod3^+/–^* mice were crossed with *Fhod3^+/–Tg(α-MHC-Fhod3CM)^* mice to obtain the rescue *Fhod3^–/–Tg(α-MHC-Fhod3CM)^* embryos. To obtain timed pregnancies, paired female mice were checked daily in the morning, and the day of vaginal plug formation was termed E0.5. Pregnant mice were sacrificed on a designated day, and embryos were dissected from the uterus followed by PCR genotyping as described previously (Kan-o *et al.*, 2012).

All experimental protocols were approved by the Animal Care and Use Committee of Miyazaki University (permit No. 2020-507-5). All mice were housed and maintained in a specific pathogen-free animal facility at the University of Miyazaki, and all efforts were made to minimize the number of animals used and their suffering. All experiments were performed in strict accordance with the guidelines for Proper Conduct of Animal Experiments (Science Council of Japan) and the Guide for the Care and Use of Laboratory Animals published by the U. S. National Institutes of Health.

### Antibodies

Rabbit anti-Fhod3 polyclonal antibodies were raised against three different regions of Fhod3, namely anti-Fhod3(650–802), anti-Fhod3(873–974), and anti-Fhod3(C-20), followed by affinity purification, as described previously (Kanaya *et al.*, 2005). The mouse monoclonal antibody against α-actinin (clone EA-53) was purchased from Sigma (St. Louis, MO, USA); the mouse monoclonal antibody against GAPDH (clone 5A12) from Wako (Osaka, Japan); Alexa Fluor 488-conjugated F(ab')_2_ fragment of anti-rabbit IgG and Alexa Fluor 555-conjugated F(ab')_2_ fragment of anti-mouse IgG from Cell Signaling Technology (Danvers, MA, USA).

### LacZ staining

LacZ staining was performed as described previously with minor modification (Sulistomo *et al.*, 2021). Briefly, the heads of embryos were fixed at 4°C by immersion in phosphate-buffered saline (PBS: 137 mM NaCl, 2.68 mM KCl, 8.1 mM Na_2_HPO_4_, and 1.47 mM KH_2_PO_4_, pH 7.4) containing 1% formaldehyde, 0.2% glutaraldehyde, 0.02% Nonidet P-40, and 1 mM MgCl_2_ followed by cryoprotection at 4°C in 30% sucrose and embedded in in OCT compound (Sakura Finetek, Tokyo, Japan). The blocks were frozen and sliced using Cryostat (CM3050S, Leica Biosystems, Nussloch, Germany) at 30 μm thickness. The sliced sections were incubated at 37°C in PBS containing 1 mg/ml 5-bromo-4-chloro-3-indolyl-β-D-galactopyranoside (X-gal), 5 mM K_3_Fe(CN)_6_, 5 mM K_4_Fe(CN)_6_, and 2 mM MgCl_2_, followed by counter staining with 1% Orange G (WAKO) in 2% phosphotungstic acid. Images were taken with a digital microscope (BZ-9000, Keyence, Osaka, Japan).

### Quantification of mRNA levels by real-time PCR

Total RNAs were extracted from the tongue tissue of wild-type C57BL/6N mice using TRIzol reagent (Invitrogen, Waltham, MA, USA). Complementary DNAs were synthesized using Superscript First-Strand Synthesis System for RT-PCR (Invitrogen). Quantitative real-time PCR were performed using SYBR Premix Ex Taq II (TaKaRa Bio, Otsu, Japan) on the QuantStudio5 (Applied Biosystems, Waltham, MA, USA). Primers for real-time PCR are listed in Supplementary Table. The gene expression level of Fhod3 was normalized to that of the housekeeping glyceraldehyde-3-phosphate dehydrogenase (GAPDH) gene or sarcomeric α-actinin (Actn2) gene.

### Detection of mRNAs for endogenous and exogenous Fhod3 by RT-PCR

Total RNAs were extracted from tongue tissue of transgenic *Fhod3^+/+Tg(α-MHC-Fhod3CM)^*, rescued *Fhod3^–/–Tg(α-MHC-Fhod3CM)^*, or wild-type embryo using RNeasy (QIAGEN, Venlo, Nederlands), and followed with TURBO DNA-free DNA removal kit (Applied Biosystems) to remove contaminating DNA. Complementary DNAs were synthesized using Superscript First-Strand Synthesis System for RT-PCR (Invitrogen). Expression of endogenous and exogenous Fhod3 was determined by PCR using specific primer sets (see Fig. 4A) and common primer sets (see Fig. 7A). The sequence of primers used are listed in Supplementary Table. Images were taken with an digital imaging system (ImageQuant LAS4000, Fujifilm, Tokyo, Japan).

### Immunoblot analysis

Immunoblot analysis was performed as described previously with minor modification (Ushijima *et al.*, 2018). Briefly, the tongues and hearts of mice were snap-frozen, crushed using SK-Mill (SK-100, Funakoshi, Tokyo, Japan), and dissolved in a buffer composed of 8 M urea, 2 M thiourea, 2% SDS, 2% Triton X-100, 1% dithiothreitol, and 10 mM Tris-HCl pH 6.8, containing Protease inhibitor cocktail (Sigma-Aldrich, St. Louis, MO, USA). The lysates were applied to SDS-PAGE and transferred to a polyvinylidene difluoride membrane (Millipore, Burlington, MA, USA). The membrane was probed with the anti-Fhod3 antibody, followed by development using ECL Prime (GE Healthcare, Chicago, IL, USA) for visualization of the antibodies. Images were taken with an digital imaging system (ImageQuant LAS4000, Fujifilm).

### Histological analysis

Histological analysis was performed as described previously (Kan-o *et al.*, 2012; Ushijima *et al.*, 2018). Briefly, timed pregnant mice were sacrificed via cervical dislocation and embryos were dissected from the uterus. The tongue (with mandibular bone) was removed from embryos under hypothermal anesthesia and then fixed by immersion in a solution containing 3.7% formaldehyde in PBS at 4°C. Fixed tissues were dehydrated in ethanol, embedded in paraffin, sectioned, and stained with hematoxylin and eosin. Images were taken with a digital microscope (BZ-9000, Keyence).

### Immunofluorescence staining

Immunostaining was performed as described previously with minor modification (Kan-o *et al.*, 2012; Sulistomo *et al.*, 2019). Timed pregnant mice were sacrificed via cervical dislocation and embryos were dissected from the uterus. The head was removed from embryos and then fixed by immersion in a solution containing 3.7% formaldehyde in PBS at 4°C. The fixed tissue were washed in PBS, subjected to osmotic dehydration overnight at 4°C in 30% sucrose, and embedded in OCT compound (Sakura Finetek). The blocks were frozen and cut into 5 μm sections using a cryostat (CM3050S, Leica Biosystems). Then sections were washed with PBS containing 0.1% Triton X-100 and blocked with a blocking buffer (Blocking One Histo, Nakalai Tesque, Kyoto, Japan) containing 2% goat serum for 10 min at room temperature. Sections were labeled overnight at 4°C with primary antibodies diluted in a dilution buffer (PBS containing 3% bovine serum albumin, 2% goat serum, and 5% Blocking One Histo), washed with PBS containing 0.1% Triton X-100, and then labeled for 1 hr at 37°C with a fluorescently labeled secondary antibody mixture in the same buffer. Images were taken with a confocal scanning laser microscope (TCS SP8, Leica Microsystems, Wetzlar, Germany).

### Statistical analysis

All data were expressed as mean ± S.D. Two groups were compared by Welch’s *t* test. A *P* value of <0.05 was considered to be statistically significant. JMP Pro 16 was used for all statistical analysis.

## Results

### The expression of *lacZ* reporter in the tongue of *Fhod3^+/–^* embryonic mice

**Fig. 1:**
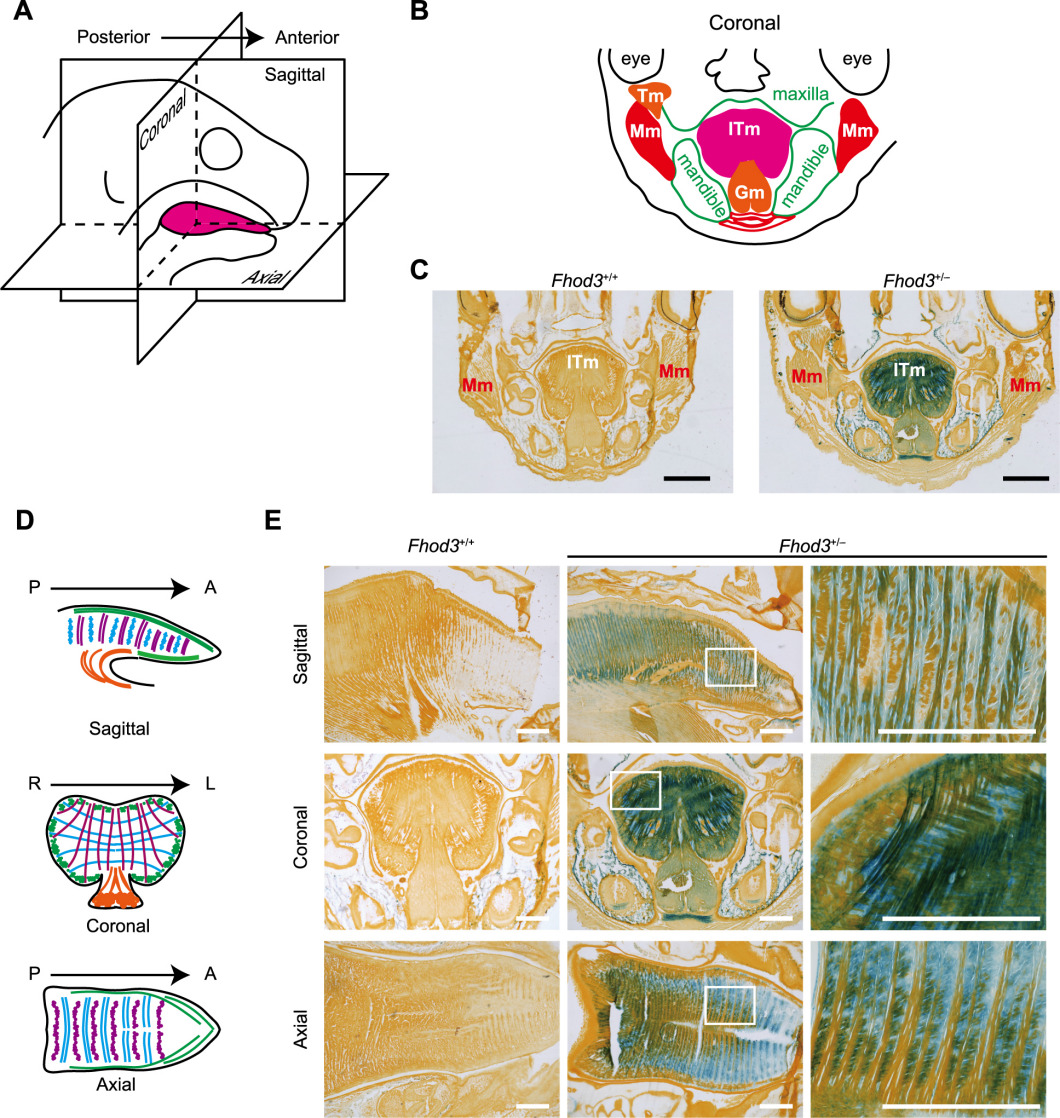
LacZ staining of the head of *Fhod3^+/–^* mice (A) Schematic representation of the anatomical planes showed in (B–E). (B) Schematic representation of anatomical structures in the coronal plane. ITm, intrinsic tongue muscle; Mm, masseter muscle; Tm, temporalis muscle; Gm, genioglossus muscle. (C) LacZ staining of the coronal section of the head of *Fhod3^+/+^* and *Fhod3^+/–^* embryos at E19.5. Scale bars, 1 mm. (D) Schematic representation of the intrinsic tongue muscles in the indicated anatomical planes. Purple, vertical muscle; blue, transverse muscle; green, superior and inferior longitudinal muscles; orange, genioglossus muscle. A, anterior; P, posterior; R, right; L, left. (E) LacZ staining of the intrinsic tongue muscles of *Fhod3^+/+^* and *Fhod3^+/–^* embryos at E19.5. Scale bars, 500 μm.

To determine the *Fhod3* gene expression in the tongue, we stained heterozygous *Fhod3^+/–^* embryonic mice, in which the first exon of the *Fhod3* gene was replaced by *lacZ* (which encodes the β-galactosidase enzyme LacZ) in one allele (Kan-o *et al.*, 2012), with the β-galactosidase substrate X-gal. This embryo was expected to express β-galactosidase under the control of the endogenous Fhod3 promoter. Coronal sections of the head (Fig. 1A) of *Fhod3^+/–^* embryos showed strong LacZ staining in the tongue, especially in intrinsic tongue muscles, whereas no LacZ staining was detected in the tongue of wild-type embryos (Fig. 1B and C). Other facial muscles, including the masseter and temporalis, showed little LacZ staining, indicating that Fhod3 is expressed specifically in the intrinsic tongue muscles. The intrinsic tongue muscles comprise vertical, transverse, and longitudinal muscles, each running in different directions and planes (Fig. 1D). Among the three types of intrinsic tongue muscle, strong LacZ staining was observed specifically in the vertical muscle (Fig. 1E), suggesting that Fhod3 may play a specific role in a particular muscle.

### The expression of mRNA for Fhod3 in the mouse tongue at various developmental stages

**Fig. 2:**
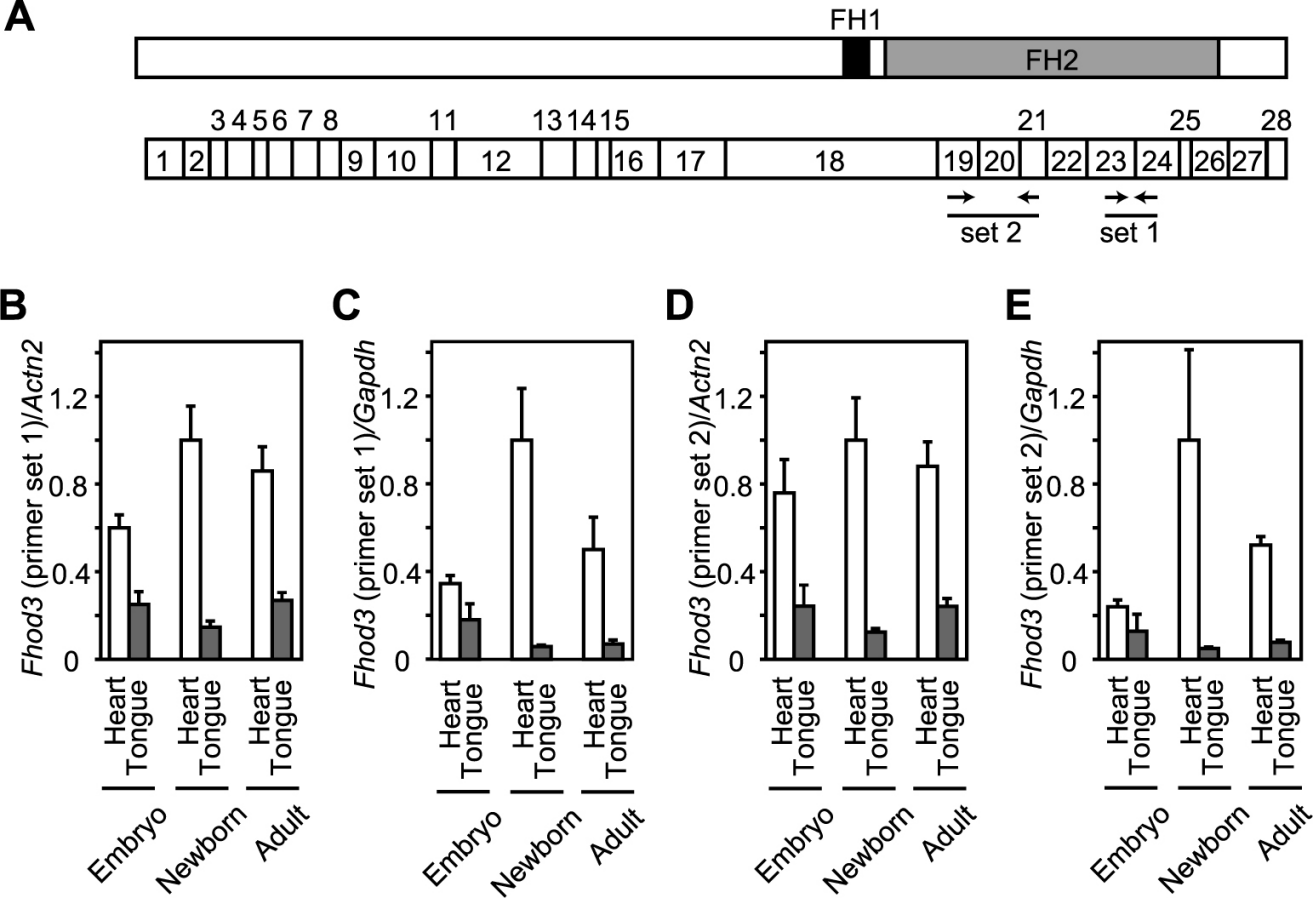
Expression of Fhod3 mRNA in the mouse tongue (A) Schematic representation of domain structure (upper) and exon structure (lower) of mouse Fhod3. Primers for RT-PCR analyses are indicated by arrows. The primer set 1 was used in (B) and (C), and the primer set 2 was used in (D) and (E). (B–E) Quantitative real-time PCR analysis of expression of Fhod3 mRNA in the tongue and heart of wild-type mice with two different pair of primers shown in (A). GAPDH (*Gapdh*) gene (C and E) or sarcomeric α-actinin (*Actn2*) gene (B and D) was used as a reference gene. Embryo, at E17.5; newborn, at P0; adult, at P28. Embryo tongue, *n* = 6; embryo heart, *n* = 4; newborn tongue, *n* = 5; newborn heart, *n* = 5; adult tongue, *n* = 3; adult heart, *n* = 4. Values are means ± SD.

Next, we performed quantitative real-time PCR to estimate the expression of Fhod3 mRNA in the tongue and heart at different developmental stages, using two different primer sets (Fig. 2A). Sarcomeric α-actinin and GAPDH were used as internal controls for RT-PCR, as the mRNA expression level of GAPDH varies across tissue types and developmental stages (Lin and Redies, 2012). As shown in Fig. 2B, a significant amount of Fhod3 mRNA was expressed in the tongue, especially during the embryonic period, although its level in the tongue was lower than that in the heart. When GAPDH was used as an internal control instead of α-actinin, the relative ratios between developmental stages and organs showed slight differences, but the basic pattern was the same (Fig. 2C). Similar results were obtained using another set of primers (Fig. 2D and E).

### The expression of Fhod3 protein in the tongue of embryonic mice

**Fig. 3:**
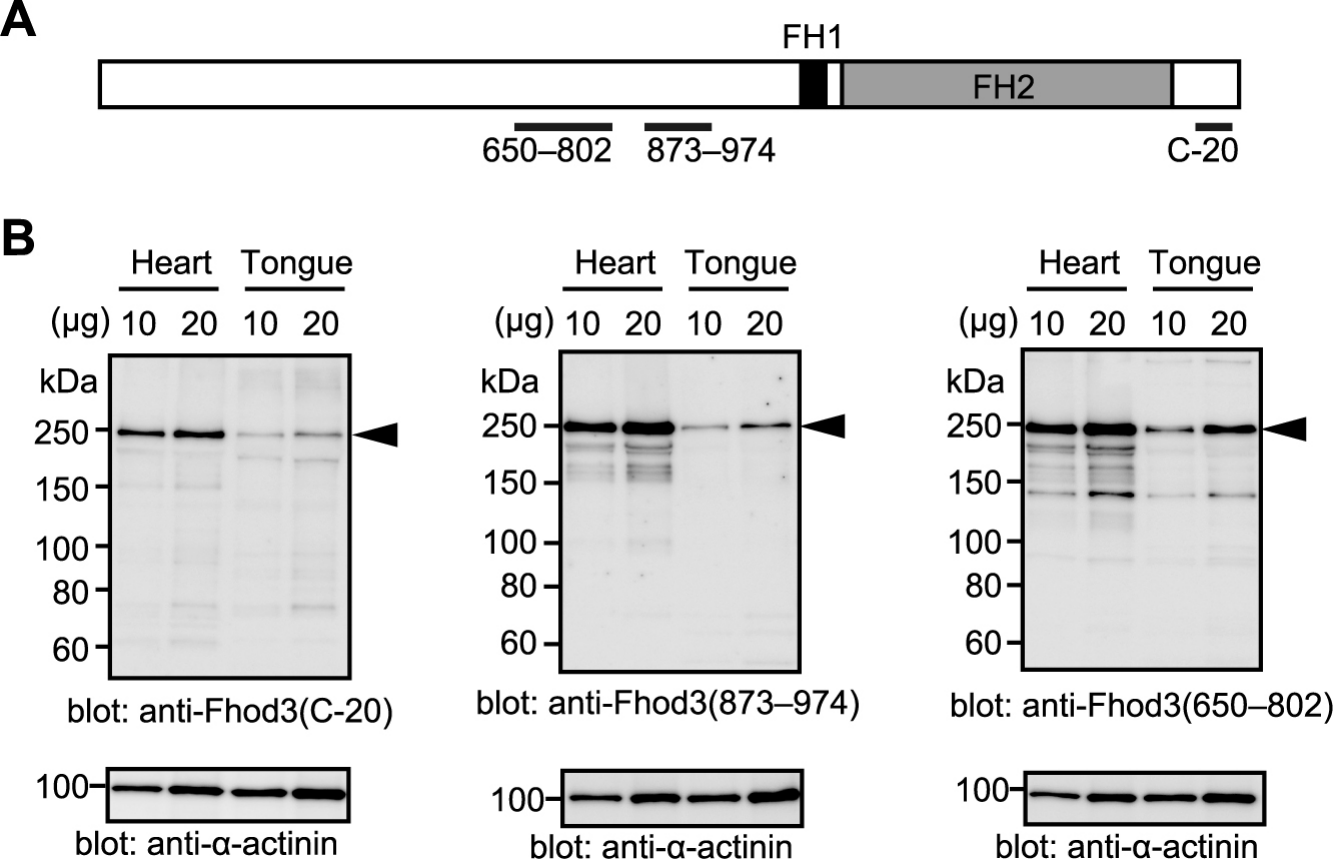
Expression of Fhod3 protein in the mouse tongue (A) Location of antigens for anti-Fhod3 antibodies used in (B). (B) Detection of Fhod3 protein by immunoblot analysis. Indicated amounts of proteins prepared from the tongue and heart of wild-type embryos at E19.5 were analyzed by immunoblot with three different anti-Fhod3 antibodies (C-20, 873–974, and 650–802) and the anti-α-actinin antibody. The arrowheads indicate the position of full-length Fhod3.

The protein expression level of Fhod3 was subsequently examined by an immunoblot analysis using three different antibodies against Fhod3, each recognizing a distinct region of Fhod3 (Fig. 3A). As shown in Fig. 3B, the Fhod3 protein was expressed in the embryonic tongue at the same size as in the heart, although the level was less than half that in the heart. Thus, Fhod3 protein was significantly expressed in the tongue, which is consistent with the mRNA expression levels estimated by quantitative real-time PCR.

### Fhod3 depletion from the embryonic tongue

**Fig. 4:**
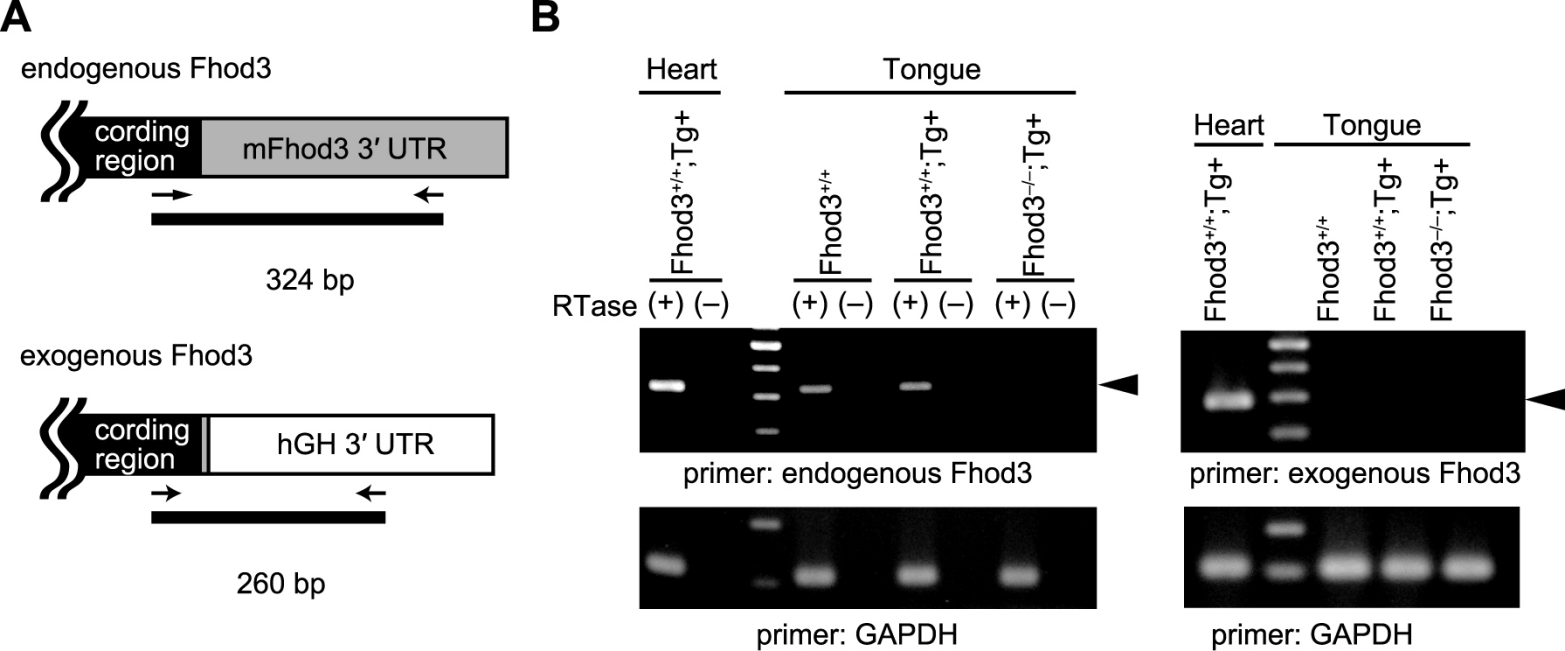
Fhod3 expression in the tongue of *Fhod3^–/–Tg(α-MHC-Fhod3CM)^* mice (A) Schematic representation of specific primers designed for detection of mRNAs for endogenous and exogenous Fhod3. The reverse primer specific for exogenous Fhod3 was designed within the 3′ UTR of human growth hormone (hGH), since the mRNA for exogenous Fhod3 contains the 3′ UTR of hGH instead of its own 3′ UTR downstream of the Fhod3 coding region. The coding region of Fhod3, the 3′ UTR of Fhod3, and the 3′ UTR of hGH are indicated by black, gray, and white boxes, respectively. (B) Expression of mRNAs for endogenous and exogenous Fhod3 in the tongue from *Fhod3^+/+^*, *Fhod3^+/+Tg(α-MHC-Fhod3CM)^*, *Fhod3^–/–Tg(α-MHC-Fhod3CM)^* embryos at E19.5. Total RNAs from embryos with indicated genotypes were treated with DNase and then subjected to RT-PCR in the presence or absence of reverse transcriptase (RTase). The arrowheads indicate the expected transcript size.

To determine the role of Fhod3 in the tongue, we depleted Fhod3 from the tongue. We previously reported that homozygous *Fhod3^–/–^* mice with defects in cardiogenesis die by E11.5 (Kan-o *et al.*, 2012), before the establishment of the tongue primordium, whereas the transgenic expression of Fhod3 in the heart under the control of the α-myosin heavy chain (α-MHC) promoter sufficiently rescues the lethality of *Fhod3^–/–^* mice by E11.5, thereby allowing embryos to develop until before birth (Kan-o *et al.*, 2012). Thus, rescued *Fhod3^–/–Tg(α-MHC-Fhod3CM)^* embryos were expected to survive until before birth and lack Fhod3 in the tongue. To confirm the absence of Fhod3 in the tongue of rescued embryos, we designed primers specific for endogenous and exogenous Fhod3 (Fig. 4A) and examined their expression in the tongue. As expected, endogenous or exogenous Fhod3 mRNA was not detected in the tongue of rescued *Fhod3^–/–Tg(α-MHC-Fhod3CM)^* embryos (Fig. 4B). This result indicates that the α-MHC promoter is active in the heart but inactive in the tongue at E19.5, and that the rescued *Fhod3^–/–Tg(α-MHC-Fhod3CM)^* embryo lacks Fhod3 mRNA in the tongue at E19.5.

### Effects of Fhod3 depletion in the tongue

**Fig. 5:**
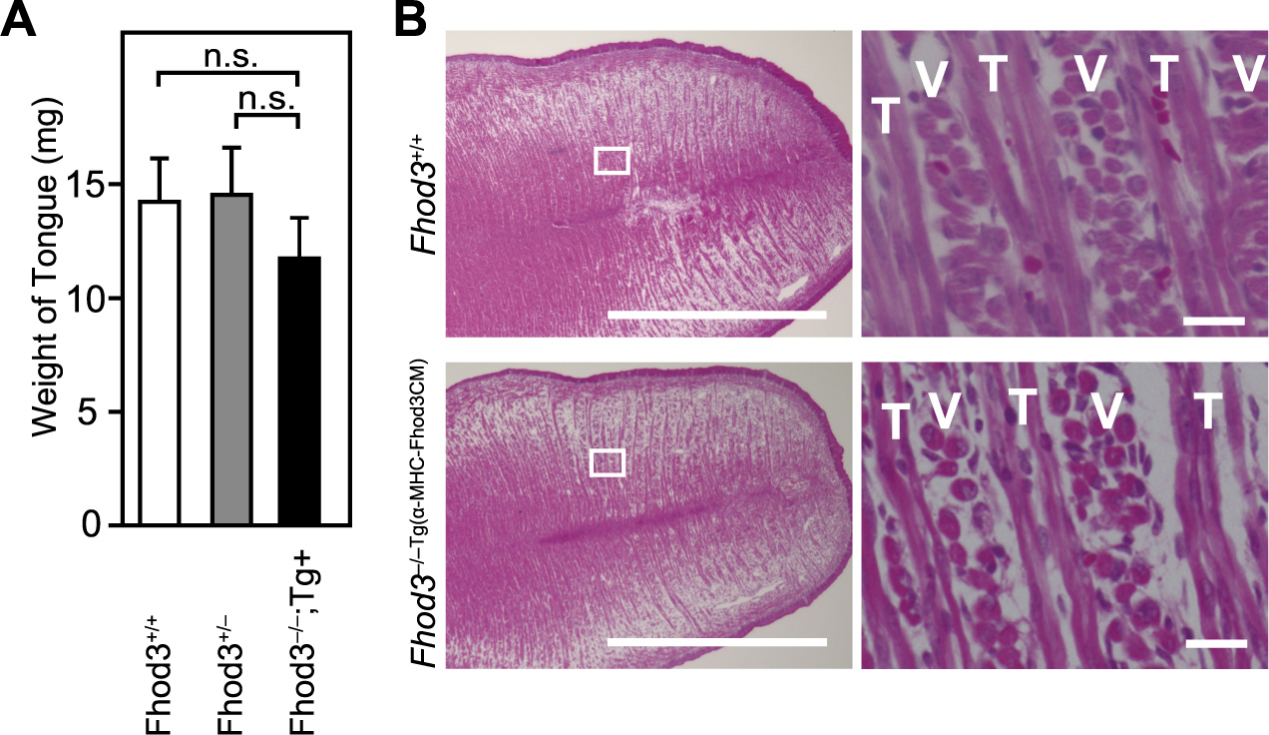
Phenotypes in the tongue of *Fhod3^–/–Tg(α-MHC-Fhod3CM)^* mice (A) Weight of the tongue of embryos at E19.5. *Fhod3^+/+^*, *n* = 4; *Fhod3*^+/–^, *n* = 3; *Fhod3^–/–Tg(α-MHC-Fhod3CM)^*, *n* = 6. Values are means ± SD. n.s., not significant (*P*>0.05). (B) Histological analysis of axial sections of tongues of *Fhod3^+/+^* and *Fhod3^–/–Tg(α-MHC-Fhod3CM)^* embryos at E19.5. V, vertical muscle; T, transverse muscle. Scale bars, 1 mm (left panels) and 20 μm (right panels).

To determine the effect of Fhod3 depletion on the embryonic tongue structure, we histologically analyzed the tongue tissue of rescue and wild-type embryos. As shown in Fig. 5A, the tongue weight of the rescued embryos appeared to be lower than that of the control embryos. The observed difference was not statistically significant, which may be attributed to the insufficient sample size. Consistent with this difference, the gross tongue size of the rescued embryos appeared to be smaller than that of the wild-type embryos (Fig. 5B, left panels). However, we did not detect clear histological differences between rescued and WT embryos (Fig. 5B, right panels), suggesting a non-essential role of Fhod3 in tongue development.

### Intracellular localization of Fhod3 protein in the tongue

**Fig. 6:**
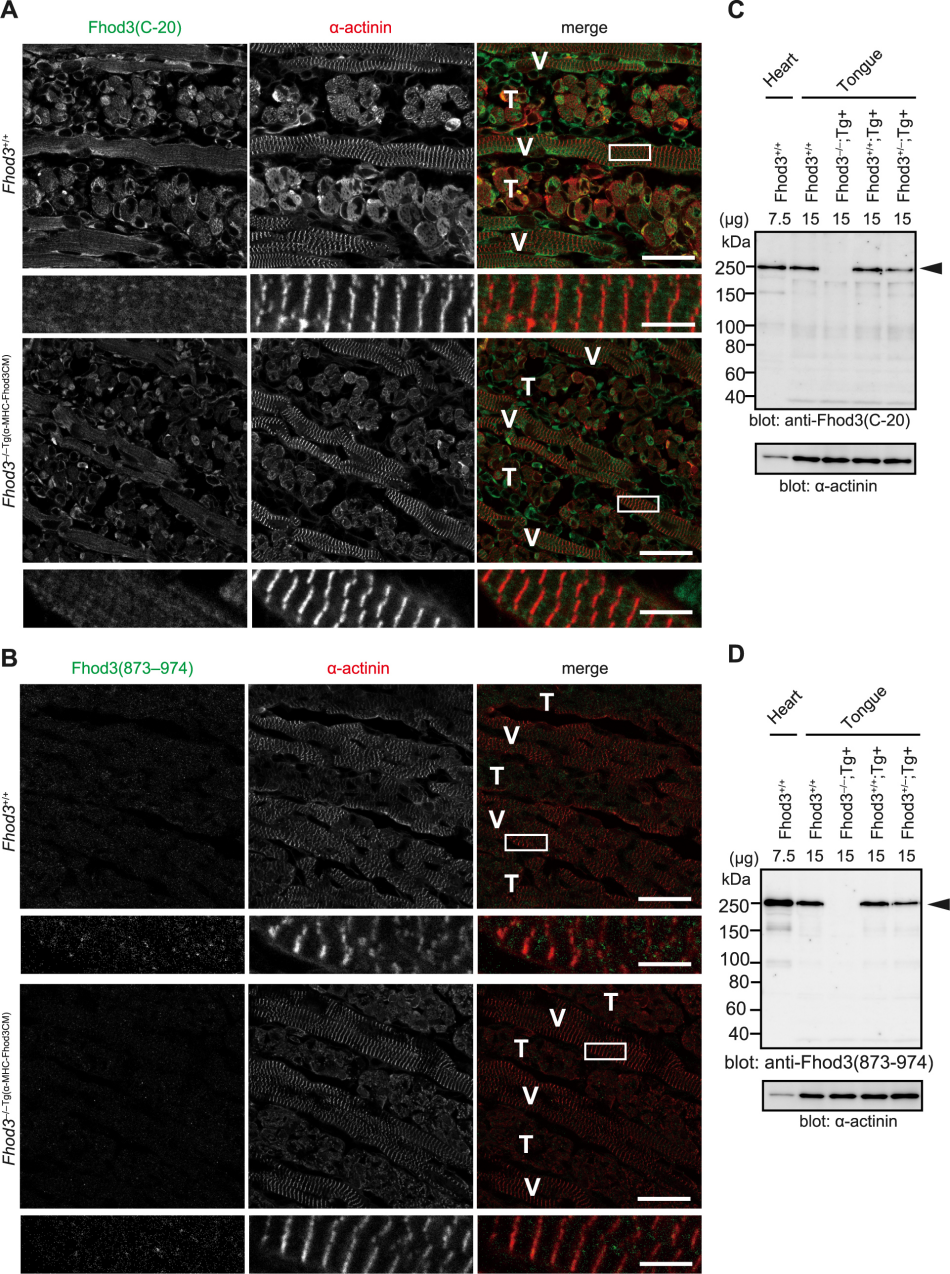
Localization of Fhod3 in the mouse tongue (A, B) Confocal fluorescence micrographs of the tongue of *Fhod3^+/+^* and *Fhod3^–/–Tg(α-MHC-Fhod3CM)^* embryos at E19.5. Sagittal sections of the tongue of embryos with indicated genotypes were immunostained with the anti-Fhod3(C-20) (A) or anti-Fhod3(873–974) (B) antibody (green), and anti-α-actinin antibody (red). These experiments have been repeated three times on three different pairs of *Fhod3^+/+^* and *Fhod3^–/–Tg(α-MHC-Fhod3CM)^* embryos with similar results. V, vertical muscle; T, transverse muscle. Scale bars, 25 μm (upper panels) and 5 μm (lower panels). (C, D) Detection of Fhod3 protein by immunoblot analysis. Indicated amounts of proteins prepared from the tongue and heart of E19.5 embryos with indicated genotypes were analyzed by immunoblot with the anti-Fhod3(C-20) (C) or anti-Fhod3(873–974) (D) antibody and anti-α-actinin antibodies. The arrowheads indicate the position of full-length Fhod3.

Next, we explored the intracellular localization of Fhod3 to address the role of Fhod3 in the tongue. In cardiomyocytes, Fhod3 localizes to the center of sarcomeres, specifically to the C-zone, and plays an essential role in regulating the assembly and maintenance of cardiac myofibrils (Matsuyama *et al.*, 2018). Therefore, we attempted to detect sarcomeric localization of Fhod3 in the tongue by immunostaining the tongue of wild-type embryos with three different antibodies against Fhod3. These antibodies have been validated for the successful detection of endogenous Fhod3 in heart tissue sections (Matsuyama *et al.*, 2018; Ushijima *et al.*, 2018). As a negative control, rescued *Fhod3^–/–Tg(α-MHC-Fhod3CM)^* embryos lacking Fhod3, which did not exhibit any differences in α-actinin localization (Fig. 6A and B) and the thin filament organization stained with phalloidin (data not shown), were used to verify the staining specificity. When we used the anti-Fhod3(C-20) and anti-Fhod3(873–974) antibodies, no significant differences were detected between tongue tissue sections from the rescued and wild-type embryos (Fig. 6A and B), although these antibodies showed clear differences between the rescued and wild-type embryos in the immunoblot analysis (Fig. 6C and D). In the tongue, while the Fhod3 expression was lower than that in the heart as detailed above, the amount of Fhod3 protein was proportional to the number of wild-type alleles and independent of the presence of the transgene: *Fhod3^+/–Tg(α-MHC-Fhod3CM)^* embryos expressed approximately half the amount of protein as *Fhod3^+/+^* embryos, while *Fhod3^–/–Tg(α-MHC-Fhod3CM)^* embryos did not express any protein, including subfragments.

On the other hand, when the immunoblot analysis was performed with the Fhod3(650–802) antibody, two strong bands of 140 kDa and 250 kDa were observed in all tongue tissues, including the rescued embryos (Fig. S1). Considering the fact that endogenous Fhod3 was not detected by the anti-Fhod3(C-20) and anti-Fhod3(873–974) antibodies (Fig. 6C and D), it is supposed that these bands detected by the Fhod3(650–802) antibody correspond to cross-reacting proteins or Fhod3 with aberrant splicing.

### The expression of alternative spliced mRNAs for Fhod3 in the tongue

**Fig. 7:**
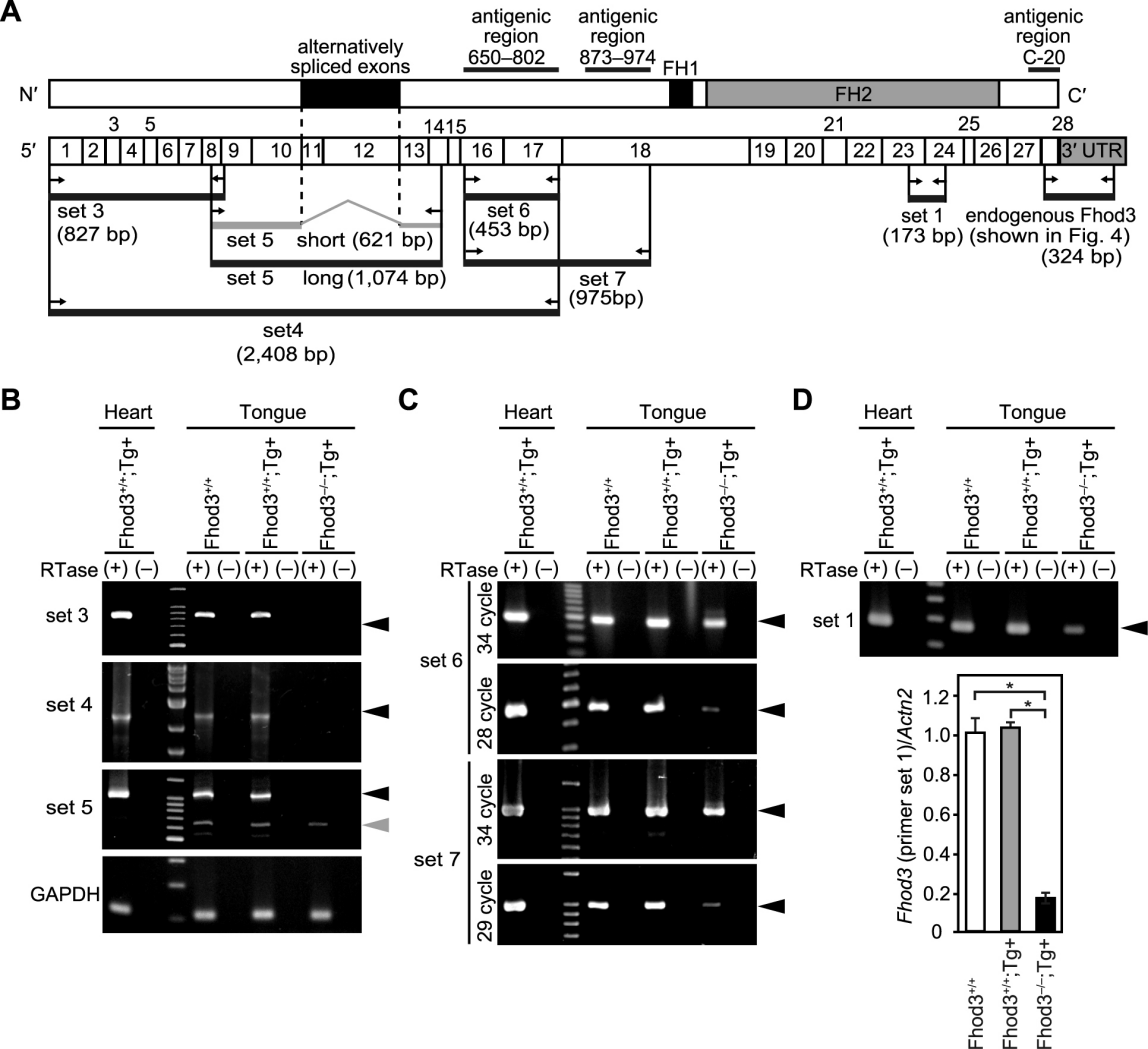
Expression of alternative spliced mRNAs for Fhod3 in the tongue (A) Schematic representation of the domain structure (upper) and exon structure (lower) of mouse Fhod3. Primers for RT-PCR analyses are indicated by arrows. The primer sets 3, 4, 5 were used in (B), the primer sets 6 and 7 were used in (C), and the primer set 1 was used in (D). Location of antigenic regions for three anti-Fhod3 antibodies are also shown. (B–D) Expression of mRNAs for Fhod3 or GAPDH in the tongue from *Fhod3^+/+^*, *Fhod3^+/+Tg(α-MHC-Fhod3CM)^*, *Fhod3^–/–Tg(α-MHC-Fhod3CM)^* embryos at E19.5. Total RNA from embryos with indicated genotypes was treated with DNase and then subjected to RT-PCR in the presence or absence of reverse transcriptase (RTase). The black and gray arrowheads indicate the expected size of the no-alternative spliced and alternative spliced transcripts, respectively. In (D), Quantitative real-time PCR analysis was conducted with the primer set 1. Sarcomeric α-actinin (*Actn2*) gene was used as a reference gene. *n* = 3 for all genotypes. Values are means ± SD. *P<0.05.

Since our knockout strategy targeted exon 1 of the *Fhod3* gene (Kan-o *et al.*, 2012), it is possible that transcripts from alternative transcription start sites are present in Fhod3-depleted tissues. In addition, aberrant splicing events were observed in transgenic mice (Davis *et al.*, 2012). We therefore examined the presence of spliced variants of Fhod3 mRNAs in the tongue. When the N-terminal primer sets 3 and 4 were used, the expression was not detected in the rescued embryo (Fig. 7B), as with the C-terminal endogenous primer set (Fig. 4B), indicating that the full-length transcript from exon1 to exon 28 is absent in the rescue embryo. However, when the primer set 5, designed to detect already known spliced variants (Kanaya *et al.*, 2005), were used, a short spliced variant was detected in the rescue embryo (Fig. 7B), indicating that this variant is probably produced from an alternative transcription start site. Since the spliced variant was detected not only in the rescue but also in wild-type embryos, it appears that this variant is not derived from the exogenous Fhod3 gene.

When the primer sets designed at antigenic regions were used, a band of the same size as that detected in the wild-type was observed in the rescue embryo, albeit in smaller quantities (Fig. 7C). Importantly, despite the presence of a transcript encoding two antigenic regions for the (650–802) and (873–974) antibodies, the protein expression level was below the limit of detection by the Fhod3(873–974) antibody (Fig. 6D), suggesting the possibility that the transcript in the rescue embryo was not expressed as a protein or expressed as an unstable protein. The low-level expression in the rescue embryo was also detected when the primer set 1 designed for quantitative PCR in Fig. 4, which is located within FH2, was used (Fig. 7D). The quantitative PCR with this primer set revealed that the expression level was less than 20% of that of the wild-type embryos (Fig. 7D). Although the identity and function of the transcript expressed at a low level in the rescue embryo have yet to be determined at this time point and require further investigation, the results of RT-PCR analysis strongly suggest that the bands detected with the Fhod3(650–802) antibody shown in Fig. S1 were considered to be cross-reacting proteins but not Fhod3 variants.

## Discussion

In the present study, we showed that Fhod3 is expressed in the tongue tissue at both the mRNA and protein levels. In comparison to the heart, the expression level was low but significant. Tongues lacking native endogenous Fhod3 showed no significant abnormalities at the histological level, suggesting that Fhod3 is not essential for tongue development. One of the objectives of this study was to gain further insight into the function of Fhod3 by elucidating its localization in the tongue. Unfortunately, the results of the present study did not provide sufficient evidence to confirm the precise localization pattern of Fhod3.

To delete Fhod3 from the tongue, we used *Fhod3^–/–Tg(αMHC-Fhod3CM)^* embryos, in which Fhod3 was systemically depleted and transgenically expressed in the heart (Kan-o *et al.*, 2012; Sulistomo *et al.*, 2021). As expected, the full-length cDNA of the Fhod3 transgene was not expressed in the tongue (Fig. 4B). Despite the absence of Fhod3 in the tongue, the rescued embryos did not show significant abnormalities in sarcomeric structure and thin filament organization, suggesting the dispensability of Fhod3 for tongue development. On the other hand, the rescued tongues were smaller than the control tongues, suggesting a general retardation of tongue development, although the difference was not statistically significant. Recently, a single-nucleus RNA-sequencing analysis of mouse skeletal muscle revealed that Fhod3 is enriched in a specific myonuclear population related to the sarcomere assembly state (Petrany *et al.*, 2020). Fhod3-mediated sarcomere assembly may occur during tongue muscle development. To determine whether Fhod3 contributes to tongue development, further studies, such as those using Wnt1-Cre-mediated conditional knockout mice, are warranted.

The vertical tongue muscles of *Fhod3^+/–^* embryos exhibited robust LacZ signals (Fig. 1). However, it should be noted that the intracellular distribution pattern of LacZ does not reflect that of Fhod3 because LacZ is a soluble non-cytoskeletal protein. To accurately determine the localization pattern of Fhod3, it is essential to conduct immunostaining of wild-type tissue and then compare the findings with those for knockout tissue. This is because of the limited specificity and affinity of the currently available anti-Fhod3 antibodies. In this context, *Fhod3^–/–Tg(αMHC-Fhod3CM)^* embryo was expected to serve as a valuable negative control for immunostaining. However, in comparison with negative control tissues, no significant differences in the immunostaining with the anti-Fhod3(C-20) and anti-Fhod3(873–974) antibodies were observed despite the fact that these antibodies were able to successfully immunostain Fhod3 in the heart.

The reasons for the discrepancy in the efficacy of these antibodies between the tongue and the heart are currently unclear. One possible reason for this is due to the fact that the antibodies were unable to effectively recognize the Fhod3 protein in the tongue tissue due to the presence of either cross-reacting proteins or unidentified binding partners. It is also possible that the signals were not recognized as specific, despite the presence of a precise antibody labeling the Fhod3 protein. If the Fhod3 protein does not show sarcomeric localization but is distributed uniformly in the cytoplasm, weak signals may not be recognized. It is also possible that Fhod3 is not uniformly distributed in a sarcomeric pattern, but is distributed only in some unexpected specific areas or populations of muscle cells. Karlsen *et al.* recently reported that Fhod3 mRNA is enriched in the myotendinous junction (MTJ), and that Fhod3 is localized at the tip of the muscle fibers in close association with the sarcolemma at the MTJ of human tissues (Karlsen *et al.*, 2023). The lingual septum or aponeurosis in the tongue muscle corresponds to the MTJ in the skeletal muscle since intrinsic tongue muscles originate from the lingual septum or aponeurosis. Therefore, we carefully observed the areas close to the lingual septum or aponeurosis, but failed to detect any specific signals in the wild-type tongue in comparison to the findings for rescued mouse tongue.

Among the various types of skeletal muscles, we focused on the intrinsic tongue muscle in this study because the LacZ signal in the intrinsic tongue muscle of *Fhod3^+/–^* embryos was much higher than that in facial muscles such as masseter and temporalis muscles (Fig. 1C). In addition, the intrinsic tongue muscles are not directly attached to the bone but run in various directions and planes, and their contraction changes the morphology of the tongue itself as a muscular hydrostat (Kier, 2012). These specific properties of tongue muscles are similar to those of the cardiac muscles (Stephenson *et al.*, 2018). Fhod3 may be involved in the specific nature of striated muscles that lack bone support. In this context, it is notable that non-vertebrate Fhod proteins play a role in the assembly of muscles lacking bone support (Shwartz *et al.*, 2016; Sundaramurthy *et al.*, 2020). On the other hand, bone-associated muscles, including the mouse tibialis anterior muscle (Petrany *et al.*, 2020) and the human hamstring muscles (Karlsen *et al.*, 2023), have also been reported to express Fhod3. Further research is required to elucidate the specific role of Fhod proteins in distinct types of striated muscle.

In conclusion, we herein report that Fhod3 is expressed in the tongue at various developmental stages. Taken together with recent findings that Fhod3 is expressed specifically in some specific populations or stages of skeletal muscles, Fhod3-mediated actin assembly seems to contribute to the development of striated muscles not only in the heart but also in non-cardiac muscles. The generation of higher-specific antibodies is eagerly awaited to expand our understanding of the physiological role of Fhod3 in striated muscles.

## Funding

This work was supported in part by JSPS KAKENHI (JP19K07355, JP22K19407, JP22H04922(AdAMS), and JP24K10079 to R.T., and JP22K11754 to Y.K., and JP21K08183 to A.M.); a bounty for pediatric cardiovascular research from Miyata Cardiac Research Promotion Foundation (to R.T.); a grant from Kobayashi Foundation (to R.T.); a grant from Suzuken Memorial Foundation (to R.T.); a grant from Kawano Masanori Memorial Public Interest Incorporated Foundation for Promotion of Pediatrics (Grant Number 35-17 to R.T.); a Grant for Clinical Research from University of Miyazaki Hospital (to H.N.); the President’s Strategic Priority Budget of the University of Miyazaki (to H.N., Y.K, and A.M.).
