# Supplementary material for: The expression of the formin Fhod3 in mouse tongue striated muscle
Source: Cell Struct Funct. 2024 Oct 10;49(2):111–22. doi: 10.1247/csf.24044 (PMC11930772; doi:10.1247/csf.24044)
Supplement: Supplementary file 1 — Supplementary Materials [file csf_49_24044_1.zip › 49_24044_Fig_S1.pdf]

Figure S1 (Nakagawa et al.)

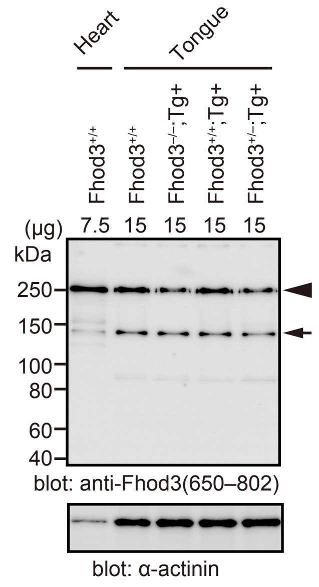

Fig. S1 Fhod3 expression detected by the anti-Fhod3(650–802) antibody

Detection of Fhod3 protein by immunoblot analysis. Indicated amounts of proteins prepared from the tongue and heart of E19.5 embryos with indicated genotypes were analyzed by immunoblot with the anti-Fhod3(650–802) antibody and anti-α-actinin antibodies. The arrowhead and arrow indicate the position of full-length Fhod3 and suspected non-specific bands, respectively.
