# Supplementary material for: The expression of the formin Fhod3 in mouse tongue striated muscle
Source: Cell Struct Funct. 2024 Oct 10;49(2):111–22. doi: 10.1247/csf.24044 (PMC11930772; doi:10.1247/csf.24044)
Supplement: Supplementary file 1 — Supplementary Materials [file csf_49_24044_1.zip › 49_24044_Supplementary_Table.docx]

**Supplementary Table**

Primer list for quantitative real-time PCR analysis.

| Gene | F/R | Sequence |
| --- | --- | --- |
| Fhod3 set 1 | Forward | 5'-GGATCCCTGTGGTCAAAACTGGAACCC-3' |
|  | Reverse | 5'-GGATTCTGCACTGCCCAACGGCACTTC-3' |
| Fhod3 set 2 | Forward | 5'-GGATCCGTGGAAAACTTTCCGGACAGC-3' |
|  | Reverse | 5'-CTCGAGCAAAACAGGCTTCATCTCGTG-3' |
| Actn2 | Forward | 5'-TGGCAGCCAGATCGAGAAC-3' |
|  | Reverse | 5'-GTGGAACCGCATTTTTTCCCC-3' |
| Gapdh | Forward | 5'-GGAAGCCCATCACCATCTTCCA-3' |
|  | Reverse | 5'-GTCTTCACCACCATGGAGAAGG-3' |

Primer list for PCR analysis shown in Fig. 4.

| Designation | F/R | Sequence |
| --- | --- | --- |
| common  (endo and exo Fhod3) | Forward | 5'-GGCACATCTGAACTGCAACTG-3' |
| endogenous Fhod3 | Reverse | 5'-GTGACAGAAGTGATTTGTTCC-3' |
| exogenous Fhod3 | Reverse | 5'-CAAGATTGTGCCACTGCACTCC-3' |

Primer list for PCR analysis shown in Fig. 7.

| Designation | F/R | Sequence |
| --- | --- | --- |
| Fhod3 set 3 | Forward | 5'-AAAGGATCCATCATGGCCACGCTG-3' |
|  | Reverse | 5'-TCCTGCCAGTGTCTTGTTCAC-3' |
| Fhod3 set 4 | Forward | 5'-AAAGGATCCATCATGGCCACGCTG-3' |
|  | Reverse | 5'-GTCTTCCTTCTCTTCCCTCTC-3' |
| Fhod3 set 5 | Forward | 5'-GTCAAACCCTGGTCCAACATC-3' |
|  | Reverse | 5'-CCTGAACGAGGATGTGAGAAG-3' |
| Fhod3 set 6 | Forward | 5'-AAATACTTGGAGCAGTTGGCA-3' |
|  | Reverse | 5'-GTCTTCCTTCTCTTCCCTCTC-3' |
| Fhod3 set 7 | Forward | 5'-AAATACTTGGAGCAGTTGGCA-3' |
|  | Reverse | 5'-AACATCGATATCGTCCTCCTC-3' |
